# Membrane Potential Assessment by Fluorimetry as a Predictor Tool of Human Sperm Fertilizing Capacity

**DOI:** 10.3389/fcell.2019.00383

**Published:** 2020-01-17

**Authors:** Carolina Baro Graf, Carla Ritagliati, Valentina Torres-Monserrat, Cintia Stival, Carlos Carizza, Mariano G. Buffone, Dario Krapf

**Affiliations:** ^1^Laboratory of Cell Signal Transduction Networks, Instituto de Biología Molecular y Celular de Rosario (IBR), CONICET-UNR, Rosario, Argentina; ^2^Laboratorio de Medicina Reproductiva, Facultad de Ciencias Bioquímicas y Farmacéuticas, Universidad Nacional de Rosario, Rosario, Argentina; ^3^Fertya, Medicina Reproductiva, Rosario, Argentina; ^4^Instituto de Biología y Medicina Experimental (IBYME), CONICET, Buenos Aires, Argentina

**Keywords:** membrane potential, sperm capacitation, *in vitro* fertilization, acrosome reaction, human infertility

## Abstract

Mammalian sperm acquire the ability to fertilize eggs by undergoing a process known as capacitation. Capacitation is triggered as the sperm travels through the female reproductive tract. This process involves specific physiological changes such as rearrangement of the cell plasma membrane, post-translational modifications of certain proteins, and changes in the cellular permeability to ions – with the subsequent impact on the plasma membrane potential (*Em*). Capacitation-associated *Em* hyperpolarization has been well studied in mouse sperm, and shown to be both necessary and sufficient to promote the acrosome reaction (AR) and fertilize the egg. However, the relevance of the sperm *Em* upon capacitation on human fertility has not been thoroughly characterized. Here, we performed an extensive study of the *Em* change during capacitation in human sperm samples using a potentiometric dye in a fluorimetric assay. Normospermic donors showed significant *Em* hyperpolarization after capacitation. *Em* values from capacitated samples correlated significantly with the sperm ability to undergo induced AR, highlighting the role of hyperpolarization in acrosomal responsiveness, and with successful *in vitro* fertilization (IVF) rates. These results show that *Em* hyperpolarization could be an indicator of human sperm fertilizing capacity, setting the basis for the use of *Em* values as a robust predictor of the success rate of IVF.

## Introduction

Infertility is a worldwide public health problem affecting ∼1 in 7, or ∼80 million couples worldwide (Slama et al., 2012; Datta et al., 2016). Although the causes of infertility are heterogeneous, the male factor is now conceived as important as the female etiology, accounting for at least 50% of cases (HFEA, 2014). Infertile couples rely on assisted reproductive technology (ART) to conceive, which include: IUI (intra-uterine insemination), *in vitro* fertilization (IVF), and ICSI (intra-cytoplasmic sperm injection). These treatments are expensive, invasive and risky. Excluding the female contribution, the decision on which treatment to choose often relies on general parameters of sperm in their basal state (i.e., non-capacitated sperm). However, these parameters are not true indicators of treatment success. Thus, a detailed understanding of how both normal and dysfunctional spermatozoa behave is necessary to develop a platform for new diagnostic tools and to infer best treatment options (Barratt et al., 2017, 2018).

Immediately after ejaculation the mammalian sperm is unable to fertilize the egg until it goes through a maturation process known as capacitation. Two capacitation events are essential for successful sperm penetration into oocytes: a vigorous motility called hyperactivation (HA) and the ability to undergo the acrosome reaction (AR) in response to a physiological agonist (Yanagimachi, 1994a, b). In addition, a series of sequential and concomitant biochemical processes must occur during capacitation, including the hyperpolarization of the plasma membrane (Zeng et al., 1995; Arnoult et al., 1999; Muñoz-Garay et al., 2001; Demarco et al., 2003; Ritagliati et al., 2018). In sperm, as in most cells, the internal ion concentrations are markedly different from those in the extracellular medium. At rest, the balance of ion fluxes, gradients, and permeabilities results in an electric potential, known as the resting *Em* (Visconti et al., 2011; Stival et al., 2016). Mammalian sperm encounter environments with very different ionic composition on their journey to meet the egg. Sperm must regulate their *Em* and adapt to the changes of external ion concentration. In turn, *Em* modulates membrane ion channels and transporters, including the sperm-specific Ca^2+^ channel CatSper and voltage-gated proton channel Hv1 (Darszon et al., 2011; Lishko et al., 2012). Ion channels and ionic gradients play key roles in orchestrating intracellular signaling pathways.

Hyperpolarization of the plasma membrane during capacitation has been thoroughly studied in mouse sperm. Initially, mouse sperm are relatively depolarized (*Em* between −35 and −45 mV) and become hyperpolarized (*Em* around −70 mV) upon capacitation (Zeng et al., 1995; Arnoult et al., 1999; Stival et al., 2015; Ritagliati et al., 2018). This hyperpolarization has been shown to be both necessary and sufficient for sperm to undergo [Ca^2+^]_*i*_ increases and the AR in response to an agonist (De La Vega-Beltran et al., 2012). Despite the different roles that membrane hyperpolarization might play during capacitation, *Em* changes have not been extensively studied in human sperm. Linares-Hernández et al. (1998) reported that the *Em* of non-capacitated sperm is around −40 mV, whereas Patrat et al. (2005) reported that capacitated sperm exhibit an *Em* of about −58 mV. On the other hand, López-González et al. (2014) used a potentiometric dye in a flow cytometry assay to show that a subpopulation of human sperm undergoes *Em* hyperpolarization upon capacitation, which correlated with an increase in intracellular pH and Ca^2+^ concentration. However, they determined relative membrane potentials through the median fluorescence observed in each condition and not absolute values. Furthermore, the physiological relevance of *Em* hyperpolarization has not been elucidated yet.

Mouse sperm lacking either the K^+^ channel Slo3 or its auxiliary subunit Lrrc52 have markedly reduced fertility (Santi et al., 2010; Zeng et al., 2011, 2015). Thus, it could be hypothesized that malfunction of K^+^ channels in human sperm might also contribute to the occurrence of subfertility in men. Brown et al. (2016) used whole-cell patch-clamp electrophysiology to assess the biophysical characteristics of sperm from men undergoing fertility treatments and compared to those from fertile, healthy donors. In approximately 10% of the samples from infertile patients there was either a negligible outward conductance or an enhanced inward current, both of which caused *Em* depolarization. Analysis of clinical data from IVF patients showed a significant association of depolarized *Em* assessed by patch-clamp with low fertilization rate (Brown et al., 2016). These results point toward the correlation of the *Em* value of a sperm sample with its fertilizing capacity. However, patch-clamp is a complex, laborious and time-consuming technique, not compatible with prediction tools needed for the clinic. In addition, in view of the heterogeneity of sperm populations, it is highly possible that values acquired by patch-clamp techniques from a few cells would not be representative of those undergoing capacitation, which could lead to incorrect conclusions.

We aimed to analyze in depth human sperm *Em* during capacitation, its role in the acquisition of HA and AR, and its association with fertilization competence. Therefore, we characterized the *Em* upon capacitation in 60 human sperm samples using a potentiometric dye in a fluorimetric assay (Baro Graf et al., 2019). Sperm from normospermic donors showed a significant *Em* hyperpolarization during capacitation. In addition, *Em* values from capacitated sperm correlated significantly with the sperm capacity to undergo induced AR, underlying the essential role of hyperpolarization in acrosomal responsiveness. More importantly, this study shows that *Em* hyperpolarization is associated with successful IVF rates. Our results pave the way for the application of *Em* measurement as a useful tool to predict the success rate of IVF procedures.

## Materials and Methods

### Ethical Approval

Volunteer donors and patients were provided with written information about the study prior to giving informed consent. The study protocol was approved by the Bioethics Committee of the *Facultad de Ciencias Bioquímicas y Farmacéuticas, Universidad Nacional de Rosario*, protocol #564/2018. The studies are in compliance with the Declaration of Helsinki principles.

### Human Sperm Preparation

Semen samples were obtained by masturbation from healthy donors after 2–5 days of abstinence and analyzed following WHO recommendations (World Health Organization, 2010). Samples that fulfilled semen parameters (total fluid volume, sperm concentration, motility, viability and morphology) according to WHO normality criteria, were considered as normospermic and those that did not fulfilled any of them as non-normospermic. Samples were allowed to liquefy for 1 h at room temperature, then, sperm ejaculates were allowed to swim-up in non-capacitating media at 37°C for 1 h. The non-capacitating medium used was HEPES-buffered human tubal fluid (HTF) containing 90.7 mM NaCl, 4.7 mM KCl, 0.3 mM KH_2_PO_4_, 1.2 mM MgSO_4_, 2.8 mM glucose, 3.4 mM sodium pyruvate, 1.6 mM CaCl_2_, 60 mM sodium lactate and 23.8 mM HEPES (pH 7.4), which was supplemented with 20 mM bicarbonate and 5 mg/ml BSA to obtain the capacitating medium. Cells were left to capacitate at 37°C as detailed for each experiment, in the same procedure as that used to prepare spermatozoa for IVF.

In Fertya (Assisted Reproduction Medical Clinic), commercially available HTF media was used for sperm preparation. The spermatozoa were separated from semen by two-layer density gradient centrifugation (Irvine Scientific Isolate) and then washed and concentrated with Quinn’s Advantage Medium with HEPES (Sage) which was supplemented with 10% Serum Protein Substitute (Sage). Washed spermatozoa were incubated to capacitate at 37°C pH 7.4 for 3–4 h prior to performing the IVF.

### Membrane Potential Assay in Cell Populations

Cells were loaded with 1 μM of the membrane-potential-sensitive dye DISC_3_(5) (Molecular Probes) for at least 5 min. No mitochondrial un-couplers were used because their contribution to the resting potential has been determined to be insignificant (Guzmán-Grenfell et al., 2000). Sperm were then transferred to a gently stirred cuvette at 37°C, and the fluorescence was monitored with a Varian Cary Eclipse fluorescence spectrophotometer at 620/670 nm excitation/emission wavelengths. Recordings were initiated when steady-state fluorescence was reached and calibration was performed at the end of each measure by adding 1 μM valinomycin and sequential additions of KCl for internal calibration curves, as previously described (Ritagliati et al., 2018; Baro Graf et al., 2019). Sperm *Em* was obtained from the initial fluorescence (measured as Arbitrary Fluorescence Units) by linearly interpolating it in the theoretical *Em* values from the calibration curve against arbitrary fluorescence units of each trace. This internal calibration for each determination compensates for variables that influence the absolute fluorescence values.

### Acrosome Status Assay

After incubation in the respective conditions, progesterone (21 μM) was added and incubated for another 30 min. Alternatively, in the NC_0_ condition, 1 μM valinomycin was added 5 min before the progesterone. Cells were seeded on eight-well glass slides. After air-drying, sperm were fixed with 3.7% formaldehyde in PBS for 15 min at room temperature, permeabilized with 0.5% Triton X-100 for 5 min, washed with PBS and incubated with PBS containing 1% BSA and FITC-conjugated pisum sativum lectin (1/200) for 1 h at room temperature. Before mounting, samples were washed with PBS (four times for 5 min each time). Epifluorescence microscopy was used to assess acrosome status. At least 200 sperm were analyzed in each condition.

### Sperm Motility Analysis

Sperm suspensions were loaded on a 30-μm deep slide and placed on a microscope stage at 37°C. Sperm movements were examined using computer-assisted semen analysis (CASA) system (IVOS Sperm Analyzer, Hamilton Thorn). Thirty frames were acquired at a rate of 60 Hz. At least 200 sperm were analyzed in each condition. The following parameters were measured: mean path velocity (VAP, μm/sec), curvilinear velocity (VCL, μm/sec), straight-line velocity (VSL, μm/sec), linearity (LIN, %), amplitude of lateral head displacement (ALH, μm), and straightness (STR, %). Sperm were considered hyperactivated when presenting VCL ≥ 150 μm/sec, LIN < 50%, and ALH ≥ 5 μm. At the time of the analysis, 0.5 mg/ml BSA was added to non-capacitated conditions to avoid sperm adherence to the slide.

### Fertilization Rate at IVF

*In vitro* fertilization was performed 3–4 h post sperm preparation in 4-well dishes (Oosafe) with 0.5 ml of Quinn’s Advantage Fertilization medium supplemented with 10% Serum Protein Substitute (Sage) and covered with 0.5 ml of tissue culture oil (Sage). In order to assure good oocyte quality, this study only involved female patients with no detected female factors. All patients were below 38 years old. One thousand sperm were incubated per cumulus-oocyte complex. A maximum of five cumulus-oocyte complex were incubated per well in K-System G185 incubator (37°C, 6.2% CO_2_, 5% O_2_) during 4 h. After this period, oocytes were denudated and incubated in individual drops in QA Protein plus Cleavage medium (SAGE^®^) covered with 0.5 ml of tissue culture oil (Sage) in the same conditions as described above. At 18 h post insemination, fertilization was analyzed with an inverted microscope. Successful fertilization was considered when two pronuclei (2PN) and two distinct or fragmented polar bodies were observed. When at least 60% of eggs were fertilized, the IVF procedure was classified as successful, according to local regulations.

### Statistical Analyses

Data are expressed as mean ± standard deviation (SD) or standard error of the mean (SEM), as indicated in each figure, of at least six independent experiments for all determinations. Statistical analyses were performed using the GraphPad Prism 6 software (La Jolla, CA, United States). Student’s *t* test was used to compare mean values between control and tested groups, while the difference between mean values of multiple groups was analyzed by one-way analysis of variance (ANOVA) with multiple comparison tests. A probability (p) value *p* < 0.05 was considered statistically significant.

## Results

### Human Sperm Hyperpolarize During Capacitation

**FIGURE 1:**
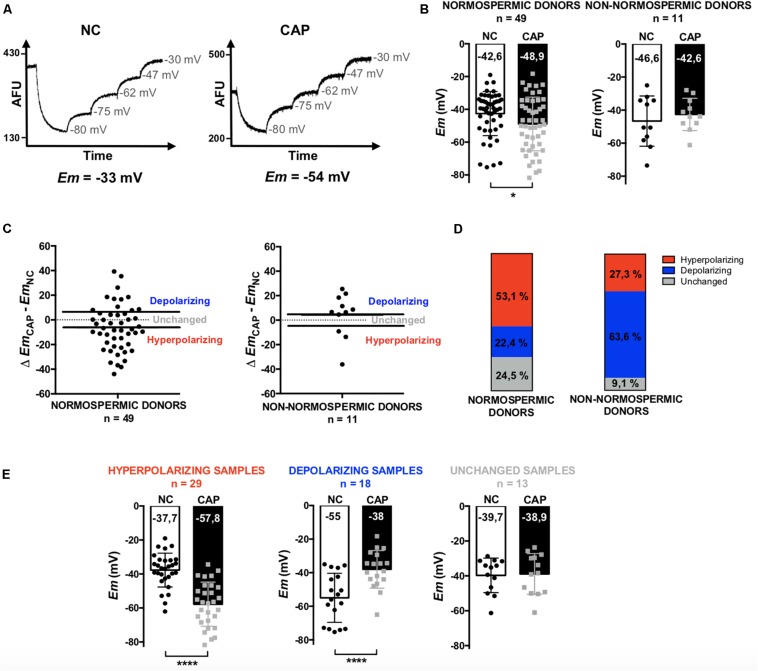
Determination of the *Em* of sperm samples by a fluorimetric assay. Non-capacitated (NC) and capacitated (CAP) human sperm from normospermic (*n* = 49) and non-normospermic (*n* = 11) donors. **(A)** Representative fluorescence traces showing *Em* values. **(B)** Distribution and mean *Em* values obtained in each condition. **(C)** Plots of the change on *Em* obtained upon capacitation (Δ *Em*_CAP_ – *Em*_NC_). The samples with a positive difference were classified as depolarizing, the ones with a negative difference as hyperpolarizing and those which did not change or with a difference under 5 mV where classified as unchanged. **(D)** Representation of the three types of behaviors among normospermic and non-normospermic donors. **(E)** Pooled *Em* values from hyperpolarizing (*n* = 29), depolarizing (*n* = 18) and unchanged (*n* = 13) samples. Data represent mean ± SD. Paired Student’s *t* test was performed between NC and CAP: ^∗^*p* < 0.05, ^****^p < 0.0001.

In this work, we performed a fluorimetric population assay to determine the absolute *Em* values of non-capacitated and capacitated sperm. When constant concentrations of sperm and probe are used, this method provides a highly reproducible value of plasma membrane potential after calibration using valinomycin (a K^+^ ionophore) and sequential additions of KCl (Baro Graf et al., 2019) (Figure 1A). A total of 60 sperm samples were analyzed, among which 49 corresponded to normospermic and 11 to non-normospermic donors, as categorized on the basis of total sperm number, ejaculate volume and motility according to WHO guidelines (see section “Materials and Methods” for details). Consistent with previous observations using flow cytometry (López-González et al., 2014), we found a large heterogeneity among *Em* values of samples from different individuals. However, normal sperm samples exhibited a significant hyperpolarization after capacitation, while sub-normal samples did not (Figure 1B). The *Em* changes (*Em*_CAP_ – *Em*_NC_) were also very variable between individuals. Both in normospermic and non-normospermic donors we could identify samples with depolarizing, hyperpolarizing or unchanged behaviors (Figure 1C). However, while in normospermic donors the majority of the samples (53.6%) hyperpolarized, among donors with sub-normal parameters, only 27.3% hyperpolarized and 63.6% depolarized (Figure 1D). When samples with different behaviors were pooled and further analyzed, *Em* changes became more evident. These results demonstrate that in most normospermic individuals there is a capacitation-induced hyperpolarization, with an initial non-capacitated *Em* value of −37.7 ± 9.9 mV, that shifts to –57.8 ± 12.9 mV upon capacitation (Figure 1E).

### Hyperpolarization Correlates With Acrosomal Responsiveness

The AR is a key step in fertilization. In mammalian sperm, the AR is mediated by intracellular calcium fluxes. Since Ca^2+^ channels in sperm cells are voltage-dependent, it has been hypothesized that gating might be controlled by *Em*.

**FIGURE 2:**
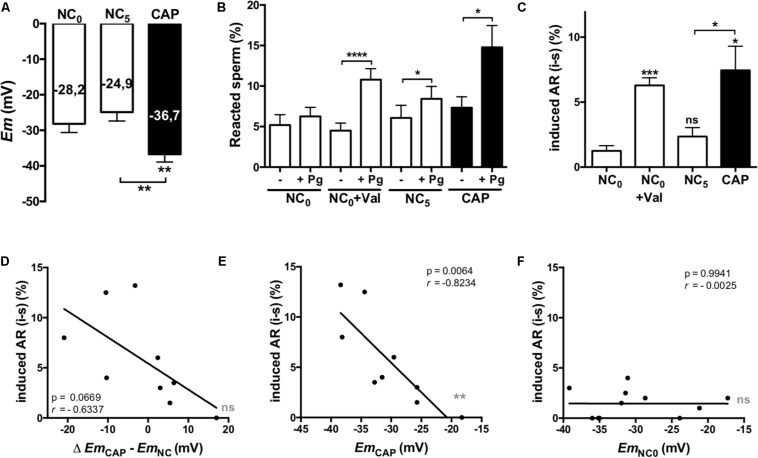
Hyperpolarized membrane potentials correlate with acrosomal responsiveness. Sperm samples from normospermic donors obtained after swim-up (NC_0_) and upon 5 h incubation in non-capacitating (NC_5_) and capacitating (CAP) media were analyzed. **(A)** *Em* measurements. **(B,C)** Cells were further incubated for 30 min in the absence (-) or presence (+) of 21 μM progesterone (Pg). The percentage of acrosome reacted sperm was assessed by FITC-PSA staining as described in section “Materials and Methods.” Data represent mean ± SEM from at least six independent experiments. Paired Student’s *t* test was performed. Statistically significant differences between the indicated conditions or with the control NC_0_ (asterisks on each column bar) are as follows: ^∗^*p* < 0.05, ^∗∗^*p* < 0.01, ^∗∗∗^*p* < 0.005, ^****^p < 0.0001. **(D–F)** Correlation analysis between the change on *Em* (**D**, Δ *Em*_CAP_ – *Em*_NC__0_) or the *Em* values in CAP **(E)** and NC_0_ **(F)** and the induced AR. The correlation coefficients (*r*) and *p* values are indicated.

We evaluated the role of the *Em* in the acquisition of acrosomal responsiveness to progesterone (Pg) in samples from normospermic donors. Both *Em* and AR were evaluated immediately after swim-up (NC_0_), as well as after 5 h in either non-capacitating (NC_5_) or capacitating (CAP) media. For AR analysis, cells were further incubated for 30 min in the absence or presence of Pg. Non-capacitated sperm obtained after swim-up (NC_0_) did not increase the percentage of acrosome reacted sperm in the presence of Pg. However, they acquired acrosomal responsiveness after pharmacological hyperpolarization with valinomycin (1 μM for 5 min) (Figures 2B,C). These results point toward the pharmacological hyperpolarization sufficiency for acrosomal responsiveness, in agreement to previous results in mouse sperm (De La Vega-Beltran et al., 2012). On the other hand, capacitated human sperm hyperpolarized (Figure 2A) and showed significantly increased induced AR (Figures 2B,C). Furthermore, a correlation analysis between *Em* change upon capacitation (*Em*_CAP_ – *Em*_NC__0_) and the induced AR (induced – spontaneous), although not statistically significant (*p* = 0.06), showed a clear negative correlation (*r* = −0.6337) (Figure 2D). Interestingly, as observed in the correlation analysis between absolute *Em* values and induced AR for each condition (Figures 2E,F), there is a strong negative correlation for sperm incubated in capacitating (*r* = −0.8234) media, but not in NC_0_ (*r* = 0.0025).

### Role of *Em* in Hyperactivated Motility

**FIGURE 3:**
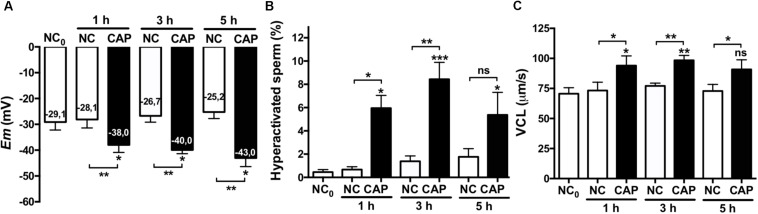
Hyperpolarization and HA. Sperm samples from normospermic donors obtained after swim-up (NC_0_) and upon incubation in non-capacitating (NC) and capacitating (CAP) media for 1, 3, and 5 h were analyzed. **(A)** *Em* measurements. **(B,C)** The percentage of hyperactivated sperm **(B)** and VCL **(C)** were obtained using CASA software. Data represent mean ± SEM from at least six independent experiments. Paired Student’s *t* test was performed. Statistically significant differences between the indicated conditions or with the control NC_0_ (asterisks on each column bar) are as follows: ^∗^*p* < 0.05, ^∗∗^*p* < 0.01, ^∗∗∗^*p* < 0.005.

Hyperactivation is essential for mammalian sperm to fertilize the oocyte (White and Aitken, 1989; Suarez et al., 1993); we therefore aimed to determine if hyperpolarization of human sperm would affect their motility. Hyperactivated motility is defined by an increase in the VCL and the ALH and a decreased LIN: VCL ≥ 150 μm/s, LIN ≤ 50% and ALH ≥ 5 μm/s, being VCL the most characteristic parameter. Figure 3 shows *Em* values (Figure 3A), percentage of hyperactivated sperm (Figure 3B) and VCL (Figure 3C) of sperm incubated in either non-capacitated or capacitated conditions at different time points. As can be observed, sperm progressively hyperpolarized in time with a maximum at 5 h (which was the longest time assayed), while the maximum HA rate and VCL were at 3 h (Figures 3A–C). In order to assess the role of hyperpolarization in sperm motility, we performed a correlation analysis of *Em* values with HA and VCL after 3 and 5 h of capacitation (Supplementary Figure S1). Though not significant, there is a tendency in the correlation between *Em* at 3 h of capacitation and VCL (Supplementary Figure S1A). On the other hand, the correlation was weaker at 5 h of capacitation (Supplementary Figure S1B), which can be explained by the fact that at 5 h sperm further hyperpolarized, while HA did not continue increasing (Figure 3). Finally, the analysis between *Em* change upon capacitation (*Em*_CAP__3_ – *Em*_NC__0_) and the VCL or HA values, showed a weak correlation (Supplementary Figure S1C).

### *Em* Analysis Predicts IVF Outcome

Aiming to further investigate the biological role of *Em* on human sperm fertilization competence, we determined the *Em* of non-capacitated and capacitated sperm from IVF patients. One important drawback in correlation analyses of human sperm is the biological variation between samples obtained from the same donors on different days. Thus, in order to avoid this issue, the fluorimetric *Em* measurements were performed on aliquots of the ejaculate used on the day of the IVF treatment, allowing direct comparison of *Em* values with fertilization success.

**FIGURE 4:**
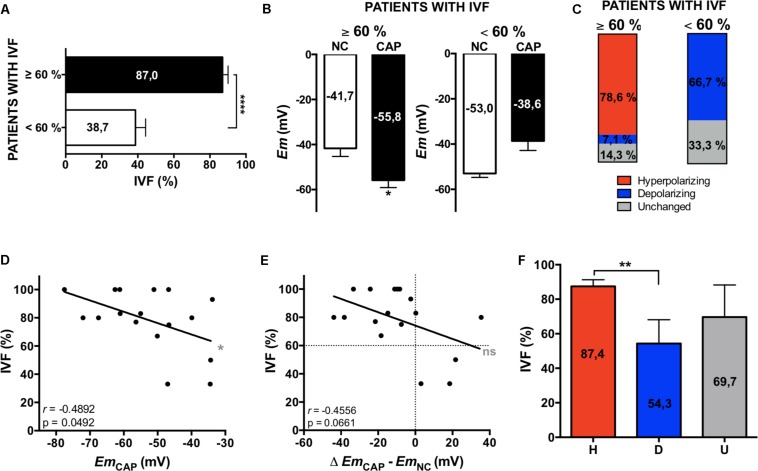
*Em* hyperpolarization correlates with higher IVF rates. **(A)** IVF patients were classified according to their fertilization rate: ≥60% and <60% of fertilized oocytes. Data represent mean ± SEM, an unpaired Student’s *t* test was performed: ^****^p < 0.0001. **(B)** *Em* measurements from patients’ samples. Data represent mean ± SEM, a paired Student’s *t* test was performed: ^∗^*p* < 0.05. **(C)** Representation of the percentages of each type of behavior: hyperpolarizing (red), depolarizing (blue) and unchanged (gray). **(D,E)** Correlation analysis between the percentage of IVF and the *Em* in CAP **(D)** and the change on *Em* (Δ *Em*_CAP_ – *Em*_NC__0_) **(E)**. The correlation coefficients (*r*) and *p* values are indicated. **(F)** Mean IVF rates of hyperpolarizing (H, red), depolarizing (D, blue), and unchanged (U, gray) samples. Data represent mean ± SEM, an unpaired Student’s *t* test was performed: ^∗∗^*p* < 0.01.

**FIGURE 5:**
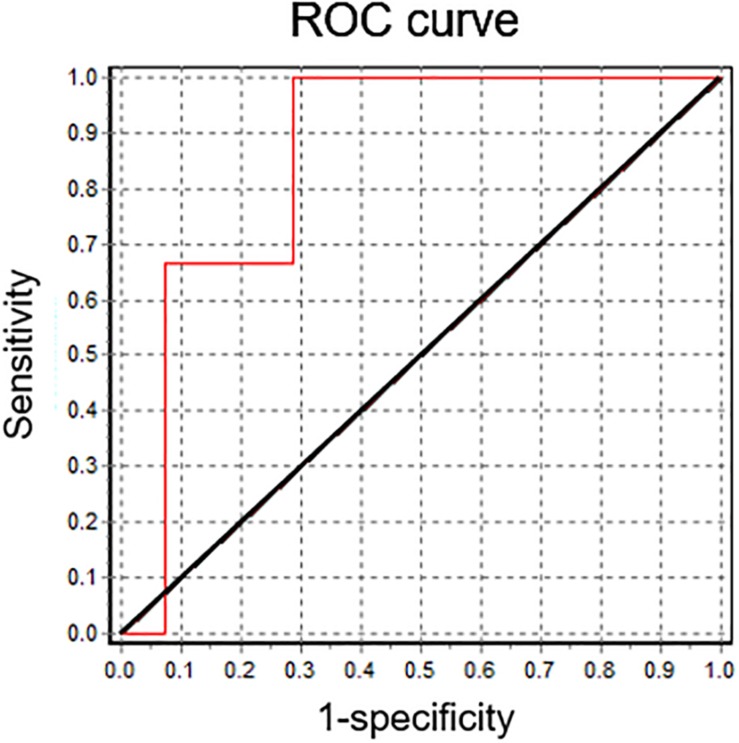
Capacitated sperm *Em* is a good predictor for IVF. Receiver operating characteristic (ROC) curve analysis for capacitated sperm *Em* values. The reference line is marked in black. The area under the curve is 0.8571 ± 0.098 (95% CI = 0.6647; 1).

*In vitro* fertilization patients were classified according to their fertilization rate, satisfying local clinical parameters for successful (fertilization rate ≥ 60%) and unsuccessful IVF procedures (fertilization rate < 60%). These two groups showed significant differences between their mean IVF success rates (Figure 4A). In a retrospective analysis, sperm samples with successful IVF rates exhibited a significant *Em* hyperpolarization during capacitation (Figure 4B). On the other hand, patients with IVF rates under 60% did not hyperpolarize (Figure 4B). As can be seen in Figure 4C, 78.6% of successful IVF samples hyperpolarized during capacitation. However, the majority of samples from poor IVF rate patients (66.7%) showed a depolarizing behavior. These results indicate that the IVF outcome might be dependent on a capacitation-associated hyperpolarization. To test this hypothesis, we performed correlation analysis of absolute *Em* values obtained after capacitation and IVF rates (Figure 4D), and also of *Em* changes upon capacitation (*Em*_CAP_ – *Em*_NC_) against IVF rates (Figure 4E). In agreement with our previous results (Figure 2) where we observed a correlation between capacitated sperm *Em* and induced AR, there was a significant correlation between capacitated sperm *Em* (*Em*_CAP_) and IVF rate. Interestingly, a predictive analysis showed that hyperpolarizing samples have significantly higher IVF rates in comparison with depolarizing samples, as depicted in Figure 4F. Finally, a ROC curve was constructed to assess the effectiveness of capacitated sperm *Em* in predicting IVF outcomes (Figure 5). The area under the curve was 0.8571 ± 0.098 (95% CI = 0.6647-1), indicating that capacitated sperm *Em* (*Em*_CAP_) is a good parameter to discriminate between successful IVF rate (>60%) and unsuccessful IVF rate (<60%). The cut off value for *Em*_CAP_ with the highest sensitivity and specificity was −48.6 mV (100% sensitivity and 71.4% specificity). Considering our results, a depolarized *Em* could be related to IVF failure in idiopathic subfertile patients.

## Discussion

A total of 60 sperm samples were analyzed, among which 49 corresponded to normospermic donors. These samples exhibited a significant *Em* hyperpolarization after incubation in capacitating media, while sub-normal samples did not hyperpolarize. This raises the question whether functional defects in the capacitation-associated hyperpolarization relate to sub-normal parameters. Although further work is needed regarding more non-normospermic donors, our results may suggest that normal semen parameters can be associated to the relative permeability of the plasma membrane, ion channels regulation and metabolic state of the sperm resulting in their ability to hyperpolarize in capacitating conditions.

The AR, a key step in fertilization, is strictly dependent on an increase in intracellular Ca^2+^ (Romarowski et al., 2016). In 1998, a voltage-dependent Ca^2+^ influx caused by sperm depolarization was described in human sperm, which was enhanced when depolarization was preceded by hyperpolarization (Linares-Hernández et al., 1998). This was consistent with a hypothesis stating that capacitation-associated hyperpolarization is required to remove voltage Ca^2+^ channels inactivation. Furthermore, the steroid hormone progesterone, which is the only well-characterized biological agonist of the AR in human sperm, activates CatSper, induces Ca^2+^ influx, membrane depolarization and AR (Aitken, 1997; Kirkman-Brown et al., 2000; Patrat et al., 2005; Lishko et al., 2011). In mouse sperm it has been shown that *Em* hyperpolarization is necessary and sufficient for cells to acquire acrosomal responsiveness (De La Vega-Beltran et al., 2012). However, in human sperm, the correlation between *Em* and acrosomal responsiveness had not been thoroughly demonstrated yet. The results presented herein indicate that *Em* hyperpolarization plays an important role in human sperm AR. We have shown that: (1) capacitated sperm exhibit significant *Em* hyperpolarization and induced AR; (2) non-capacitated depolarized sperm only gain acrosomal responsiveness after pharmacological hyperpolarization; and (3) there is a strong and significant negative correlation between *Em* and induced AR in capacitated sperm. Altogether, these data support an important role of *Em* hyperpolarization for the acquisition of acrosomal responsiveness.

It is generally accepted that good sperm motility is a central component of male fertility. Individuals with poorly motile or immotile sperm are considered infertile or subfertile, and in need of ART procedures. In fact, asthenozoospermia is the commonest problem underlying male subfertility (Van Der Steeg et al., 2011) and because the root cause of this condition is usually not known, treatments for this problem are non-specific. In this context, we aimed to understand whether human sperm *Em* hyperpolarization and HA were linked. A correlation tendency was observed at 3 h between both parameters. It is well established that intracellular calcium plays a pivotal role in sperm motility regulation. Alasmari et al. (2013) showed that defects in Ca^2+^ signaling lead to poor HA and that the ability to undergo Ca^2+^ -induced HA affects sperm fertilizing capacity. This is in agreement with our results and with the hypothesis that the *Em* might play a role in regulating Ca^2+^ channels and, consequently, in intracellular calcium and indirectly in sperm motility (reviewed in Ritagliati et al., 2018).

Regardless the amount of work invested toward understanding the molecular basis of sperm capacitation, studies of male factor issues attending reproductive clinics seem to be completely dissociated from basic science knowledge. Regretfully, semen diagnostic analysis hardly involves sperm function evaluation, i.e., capacitation parameters required for fertilization. With this in mind, we aimed to analyze whether *Em* hyperpolarization relates to human sperm fertilizing capacity. It has been recently proposed that a certain degree of *Em* shift is associated with normal sperm function, as assessed by electrophysiology and IVF outcome (Brown et al., 2016). However, this study involved laborious techniques that hampered the analysis of many cells per patient. Thus, we pursued the study of sperm *Em* from patients attending a fertility clinic using a robust and relatively simple technique. In the fluorimetric population assay, the sample’s behavior is followed throughout the experiment and is performed in the physiological working conditions allowing more accurate correlations (Baro Graf et al., 2019). *Em* measurements were performed on aliquots of the same samples used in IVF procedures. Our data show that almost 80% of successful IVF samples hyperpolarized. On the other hand, there was no hyperpolarization in samples with unsuccessful IVF rates. In fact, the majority of these samples (66.7%) depolarized during incubation in capacitating conditions. Accordingly, there is a significant strong correlation between capacitated sperm *Em* and IVF rate. These data strongly suggest that human sperm *Em* changes have an implication in sperm fertilizing capacity. Interestingly, in a predictive study, samples that depolarized upon capacitation exhibited lower fertilization rates. After a ROC analysis we determined that the *Em* absolute value from capacitated sperm can be considered as a good parameter to predict IVF rates, with a cut-off value of −48.6 mV. Although further work is needed in order to increase the number of patients analyzed, and to evaluate whether this technical approach could also predict IUI success, this study has the potential to add diagnostic tools to help predict the success of reproductive techniques.

During preparation of this manuscript, we contacted the group of Dr. Celia Santi at Washington University School of Medicine in Saint Louis, who independently achieved the same results using a different methodology. Both this manuscript and Santi’s work are intended to be published concurrently (Puga Molina et al., 2020).

## Data Availability Statement

All datasets generated for this study are included in the article/Supplementary Material.

## Ethics Statement

The studies involving human participants were reviewed and approved by the Bioethics Committee of the Facultad de Ciencias Bioquímicas y Farmacéuticas, Universidad Nacional de Rosario. The patients/participants provided their written informed consent to participate in this study.

## Author Contributions

CB, CR, and CS conducted the experiments. VT-M and CC designed and conducted the IVF procedures. CB, CR, MB, and DK conceived the study. CB, CR, and DK wrote the manuscript. All authors analyzed the data and revised the final version of the manuscript.

## Conflict of Interest

The authors declare that the research was conducted in the absence of any commercial or financial relationships that could be construed as a potential conflict of interest.
