# Supplementary figures and images for: Membrane Potential Assessment by Fluorimetry as a Predictor Tool of Human Sperm Fertilizing Capacity
Source: Front Cell Dev Biol. 2020 Jan 17;7:383. doi: 10.3389/fcell.2019.00383 (PMC6979052; doi:10.3389/fcell.2019.00383)

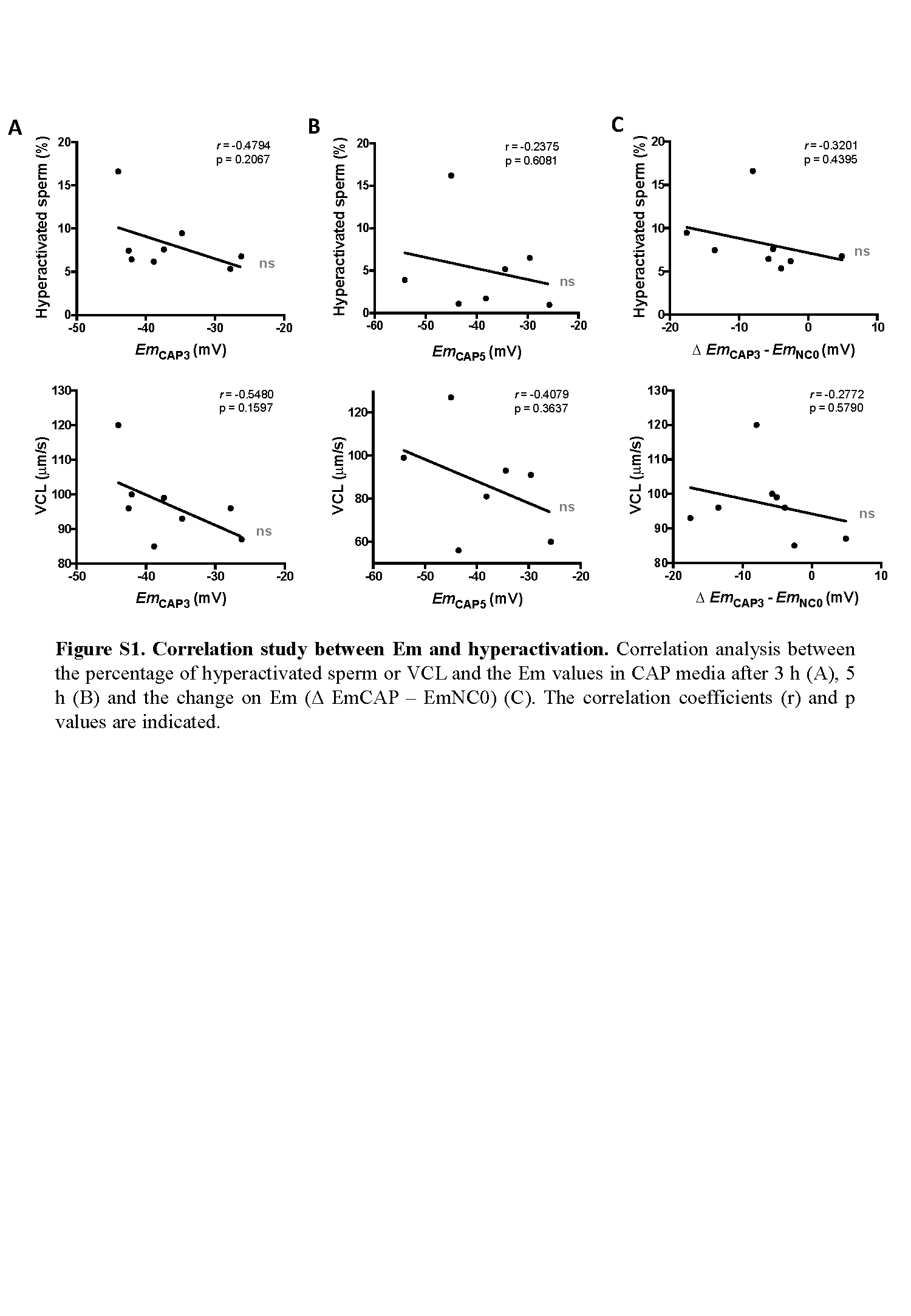

Supplement: Supplementary file 1 [file Image_1.TIFF]
